# *Nocardia* Osteomyelitis in Humans—A Narrative Review of Reported Cases, Microbiology, and Management

**DOI:** 10.3390/pathogens14101032

**Published:** 2025-10-12

**Authors:** Afroditi Ziogou, Alexios Giannakodimos, Ilias Giannakodimos, Stella Baliou, Andreas G. Tsantes, Petros Ioannou

**Affiliations:** 1 Department of Medical Oncology, Metaxa Cancer Hospital of Piraeus, 185 37 Piraeus, Greece; 2 Department of Cardiology, Tzaneio General Hospital of Piraeus, 185 37 Piraeus, Greece; 3 Department of Urology, Attikon General Hospital of Athens, 124 62 Athens, Greece; 4 School of Medicine, University of Crete, 71003 Heraklion, Greece; 5 Laboratory of Hematology and Blood Bank Unit, “Attikon” University Hospital, School of Medicine, National and Kapodistrian University of Athens, 124 62 Athens, Greece

**Keywords:** *Nocardia*, nocardiosis, osteomyelitis, bone infection

## Abstract

Nocardiosis is an infection caused by Gram-positive, saprophytic bacteria most often affecting immunocompromised hosts. The lungs, central nervous system, and skin are the sites most typically involved, although any organ may be affected. Skeletal involvement, particularly osteomyelitis, remains uncommon. This study is a review of all published cases of *Nocardia* osteomyelitis in humans, emphasizing epidemiology, microbiology, clinical features, management, and patient outcomes. A narrative review was performed using data from the PubMed/MedLine and Scopus databases. Fifty studies describing 55 patients were included. The median age was 54 years, and 65.5% were male. The main risk factors were immunosuppression (21.8%) and trauma (18.2%). The vertebrae constituted the most commonly affected site (25.5%), followed by the lower limb bones (20%); 23.6% had multifocal disease. *Nocardia asteroides* accounted for the majority of cases (34.8%). Trimethoprim-sulfamethoxazole was the most frequently administered agent (81.5%), followed by cephalosporins (29.6%) and carbapenems (27.8%). Overall mortality was 9.3%, with 5.6% of reported deaths directly attributed to the infection. Although uncommon, osteomyelitis due to *Nocardia* spp. should be considered when Gram-positive, filamentous microorganisms are detected in bone specimens, particularly in immunocompromised or post-trauma patients, as early suspicion and targeted therapy may improve survival.

## 1. Introduction

*Nocardia* species are Gram-positive bacteria with a filamentous, branching rod-like structure. This microorganism is widespread in nature, particularly in soil, and is most commonly acquired through the inhalation of contaminated environmental particles [1]. While it predominantly causes disease in people with weakened immune systems, it is estimated that as many as one-third of documented infections occur in individuals with normal immune function [2]. Nocardiosis may present as an acute, subacute, or chronic disease with a wide clinical spectrum; lower respiratory infection is the most frequently reported, accounting for approximately 62–80% of cases [2,3,4]. Other manifestations include skin involvement, typically following direct traumatic inoculation as well as infection of the central nervous system (CNS). Especially in individuals with compromised immunity, *Nocardia* spp. may disseminate via the bloodstream, leading to secondary involvement of the brain and bones [5,6]. Bone involvement, such as osteomyelitis, is uncommon, and only a limited number of cases of osteomyelitis caused by *Nocardia* spp. have been reported in the current literature, highlighting the need for further research to support the timely recognition and management of this rare yet life-threatening condition. A major diagnostic challenge lies in its frequent misidentification as tuberculosis, actinomycosis, or even malignancy, which can delay appropriate treatment. The early recognition of nocardial osteomyelitis is critical, as timely diagnosis and targeted therapy can significantly improve the patient outcomes.

The present narrative review aims to examine all documented cases of osteomyelitis provoked by *Nocardia* species, with particular emphasis on epidemiology, clinical manifestations, microbiological characteristics, therapeutic strategies, and patient outcomes. This review is motivated by the rarity and diagnostic challenges of nocardial osteomyelitis, particularly in immunocompetent patients. Given the limited and fragmented data currently available, it seeks to bridge these gaps by synthesizing existing case reports and case series to provide a clearer understanding of this condition.

## 2. Materials and Methods

### 2.1. Search Strategy and Inclusion and Exclusion Criteria

This narrative review was conducted following a predetermined protocol that was approved by all contributing authors. The primary objective was to compile and present all of the existing literature on human cases of osteomyelitis caused by *Nocardia* species while also evaluating the associated mortality rates and epidemiological patterns. A structured, directed literature search was designed to support a descriptive narrative synthesis rather than a systematic review. Therefore, the PRISMA guidelines were not applied. Furthermore, the review aimed to identify the anatomical sites of infection, describe the clinical characteristics of affected individuals as well as summarize the microbiological findings along with therapeutic approaches for *Nocardia* spp. osteomyelitis. One reviewer (A.Z) screened records with verification by a second reviewer (A.G.) for obviously eligible items using the PubMed/Medline and Scopus databases and covering all records up to 10 August 2025. The search strategy followed predefined terms: “*Nocardia*” AND (“osteomyelitis” OR “bone”). Disagreements were resolved by discussion with a senior reviewer (P.I.). Studies were selected based on their clinical relevance and scope. Specifically, original research articles, such as case reports or case series, were prioritized when they focused on the epidemiology and clinical outcomes of *Nocardia* spp. osteomyelitis in humans. Only English-language publications were considered as a feasibility measure due to resource limitations in screening non-English literature.

Publications were not considered if they were systematic or narrative reviews summarizing aggregated data, animal research or studies without full-text access, or with insufficient details on mortality or epidemiology. Additionally, to maximize completeness, the reference lists of all included articles were screened for potentially relevant publications that were not retrieved during the initial literature search.

### 2.2. Data Extraction and Definitions

From each study, data were extracted on publication year, study type, country of origin, and patient demographics including age or sex along with relevant medical history, microbiological results, and infection characteristics. The latter included the particular affected bone, diagnostic methods, complications, causative pathogens, antimicrobial resistance patterns, treatment regimens, and clinical outcomes, noted as survival or death. Mortality was attributed to the initial infection when explicitly stated by the original authors.

## 3. Results

### 3.1. Characteristics of the Included Studies

**Figure 1:**
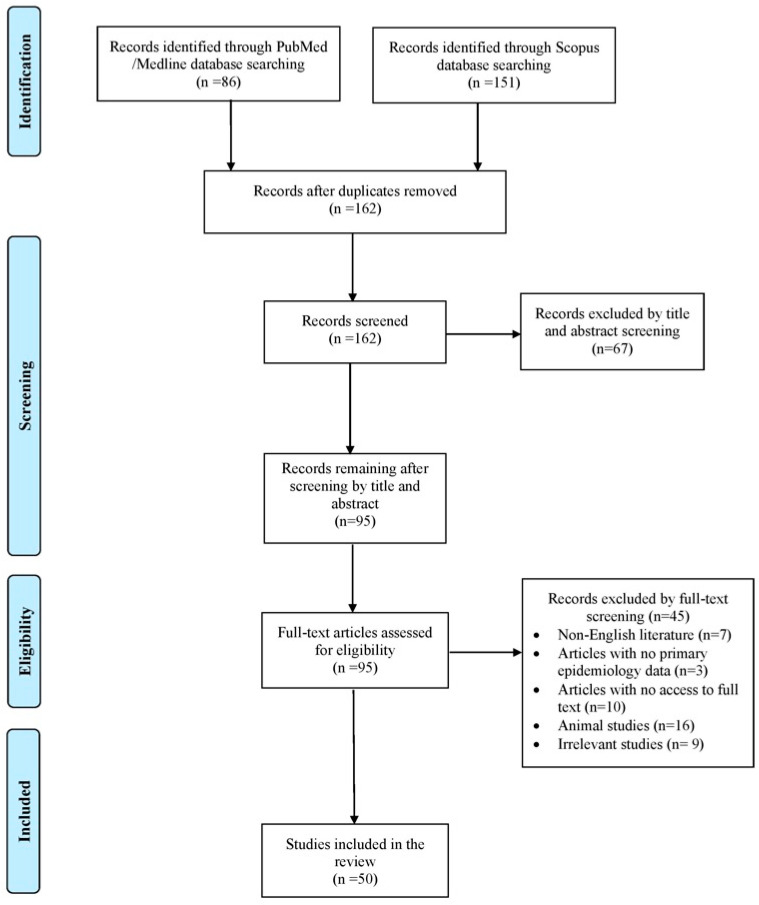
Search and selection flow of this review.

The database search yielded 237 records from PubMed/Medline and Scopus. After removing duplicates, screening titles and abstracts, and applying citation chasing, 50 studies met the inclusion criteria and were included for full review, representing 55 individual cases [3,5,6,7,8,9,10,11,12,13,14,15,16,17,18,19,20,21,22,23,24,25,26,27,28,29,30,31,32,33,34,35,36,37,38,39,40,41,42,43,44,45,46,47,48,49,50,51,52,53]. The selection process is illustrated in Figure 1.

**Figure 2:**
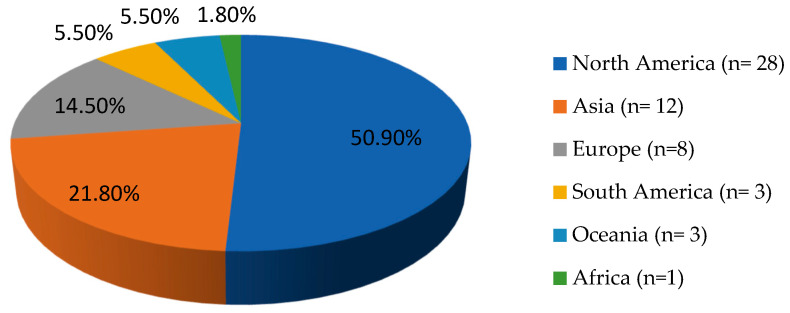
Geographical distribution of *Nocardia* spp. osteomyelitis cases worldwide (*n* = 55).

The included studies contained 48 case reports and 2 case series. Among these cases, most originated from North America, followed by Asia and Europe. The exact geographical distribution of all *Nocardia* osteomyelitis cases is illustrated in Figure 2.

### 3.2. Epidemiology of Nocardia spp. Osteomyelitis

The median age of patients with *Nocardia* spp. osteomyelitis was 54 years, varying from 1 to 87 years, with males accounting for 65.5% 36/55). Concerning medical history and risk factors, 12/55 patients (21.8%) were immunosuppressed, and 10/55 (18.2%) had a history of trauma. History of recent surgical procedures and antibiotic administration were observed in 7/55 patients (12.7%) each, whereas 5/55 individuals (9.1%) had a history of organ transplantation. The most commonly transplanted organ was the kidney in three out of these five patients (60%). A history of diabetes mellitus and autoimmune syndrome were documented in 4/55 patients (7.3%), respectively, while neutropenia was reported in 3/55 cases (5.5%). Two cases (3.6%, 2/55 patients) of HIV infection and malignancy were reported, respectively; the latter included only solid organ tumors. History of tuberculosis and chronic renal disease were identified in 1/55 case each (1.8%). Notably, in 16/55 cases (29.1%), no predisposing factors could be identified. A comprehensive summary of the demographic and clinical features of *Nocardia* osteomyelitis cases is presented in Table 1.

### 3.3. Microbiology and Antimicrobial Resistance of Nocardia Osteomyelitis

*Nocardia* spp. was detected in bone cultures from 27/54 patients (50%). In 22/54 individuals (40.7%), the pathogen was isolated from pus cultures derived from abscess drainage. In a smaller subset of cases, the organism was isolated from other sources: sputum cultures or bronchoalveolar lavage in 2/54 cases (3.7%), and ear swab, sinus secretions, and blood culture in 1/54 patient (1.9%), respectively. Of note, *Nocardia* spp. was isolated from a variety of biological samples, combinations of bone, tissue, or pus cultures in 11/54 patients (20.4%, with the available data). Among the identified isolates, *Nocardia asteroides* was the predominant species, found in 16/46 patients (34.8%), followed by *N. farcinica* in 8/46 cases (17.4%), and *N. brasiliensis* and *N. nova* each in 4/46 cases (8.7%). Other species rarely detected included *N. cyriacigeorgica* in 3/46 patients (6.5%), and *N. veterana*, *N. abscessus*, and *N. otitidiscaviarum* in 2/46 patients (4.4%). Finally, *N. asiatica*, *N. transvalensis*, *N. vulneris*, *N. madurae*, and *N. pseudobrasiliensis* were each identified in 1/46 patients (2.2%). *Nocardia* spp. identification most frequently relied on histopathological examination, exploiting the pathogen’s distinctive microbiological features. Advanced molecular techniques were employed only in 13/55 cases, with DNA gene sequencing and 16s rRNA used in 6/13 (46.2%) and 5/13 (38.5%) patients, respectively. Matrix-assisted laser desorption/ionization time-of-flight mass spectrometry (MALDI- TOF MS) was applied in 2/13 cases (15.4%).

Concomitant infections were recorded in 5/55 patients (9.1%) and included one case of urinary tract infection (UTI), one case of bacteremia, one case of both UTI and bacteremia, one case of upper and one of lower respiratory infection. In two of these cases, *Staphylococcus aureus* was the identified co-pathogen. Polymicrobial infection was reported in 7/55 individuals (12.7%), with *Staphylococcus* spp. being the most commonly co-isolated pathogen in 4 of these cases.

Antimicrobial susceptibility testing was performed in only 24/55 patients (43.6%). Resistance was relatively common, detected in 18/24 cases (75%). Table 2 summarizes the antimicrobial resistance profiles. The spectrum of antibiotics tested varied across patients, with each case assessed against a distinct combination of drugs. Trimethoprim/sulfamethoxazole (TMP/SMX) and linezolid were most frequently subjected to susceptibility testing. Notably, in all cases where vancomycin susceptibility was evaluated, the isolates demonstrated sensitivity.

### 3.4. Clinical Presentation of Nocardia spp. Osteomyelitis

The vertebrae represented the most frequent site of infection, identified in 14/55 individuals (25.5%). The next most commonly affected structures were the bones of the lower limbs, tibia or fibula, reported in 11/55 patients (20%). Involvement of the skull or femoral bone was recorded in 8/55 cases (14.5%) each, while the bones of the feet and pelvic bones were affected in 6/55 (10.9%) and 5/55 (9.1%) patients, respectively. Infection of the thoracic bones occurred in 4/55 cases (7.3%), and the bones of the hands were implicated in 3/55 cases (5.5%). Less often, osteomyelitis was found in the upper extremity bones, reported in 2/55 patients (3.6%), whereas patellar and zygomatic bone involvement appeared in only one case (1.8%). Notably, multifocal osteomyelitis was documented in 13/55 patients (23.6%).

Primary osteomyelitis was observed in 41/55 patients (74.5%); concerning the remaining 14 cases, the infection was secondary from other infected sites such as the lungs or septic arthritis. Beyond bone structures, cutaneous infection was seen in 30/55 cases (54.5%). The lower respiratory tract was affected in 11/55 patients (20%), the central nervous system (CNS) in 9/55 patients (16.4%), while 3/55 individuals developed bacteremia (5.5%). Two cases (3.6%) of upper respiratory infection were recorded. Regarding co-existing UTI, gastrointestinal infection, or endocarditis due to *Nocardia* spp., only one case was documented, respectively (1.8%).

Concerning clinical complications, abscesses constituted the predominant clinical manifestation, occurring in 31/55 patients (56.4%). Fever followed closely, observed in 25/55 cases (45.5%), while fistula formation was present in 16/55 patients (29.1%). Ulcerative lesions developed in 7/55 cases (12.7%), and bone fractures were reported in 6/55 patients (10.9%). Sepsis or organ dysfunction occurred in 5/55 patients (9.1%), while renal failure was observed in 3/55 cases (5.5%). Intensive care unit admission was necessary for only one patient (1.8%). Interestingly, amongst the included cases, no patient developed septic shock.

Rates of misdiagnosis were notable (29.1%), with 50% of cases misdiagnosed as infections and 25% as immune-mediated conditions.

### 3.5. Treatment and Outcome of Nocardia spp. Osteomyelitis

Among the documented cases, antimicrobial treatment was administered to 54/55 patients (98.2%). For one patient, it was unclear whether antibiotics were administered. TMP-SMX represented the predominant agent, prescribed in 44/54 instances (81.5%). Cephalosporins were the next most frequently used, given to 16/54 patients (29.6%), while carbapenems were prescribed in 15/54 cases (27.8%). Aminoglycosides and quinolones were administered in 11/54 (20.4%) and 9/54 (16.7%) patients, respectively, while 8/54 patients (14.8%) received tetracyclines. Linezolid and aminopenicillins were less common, being used in 7/54 (13%) and 6/54 (11.1%) patients, respectively. Only 4/54 patients (7.4%) received vancomycin. Notably, a combination of antibiotics was administered to 45/54 patients (83.3%). In 26/54 cases (48.2%), antibiotics were given empirically and treatment was adjusted after pathogen identification. Surgical intervention accompanied medical management in 41/55 patients (74.5%), most often consisting of debridement of the infected bone and abscess drainage. The median treatment duration among survivors was 10 months. Overall mortality reached 5/54 cases (9.3%), with 3 of these deaths (5.6%) directly attributed to *Nocardia* osteomyelitis.

## 4. Discussion

This narrative review investigated cases of osteomyelitis attributed to *Nocardia* species, synthesizing data from diverse reports to provide an in-depth overview of their epidemiological patterns, microbiological profiles, clinical characteristics, management strategies, and outcomes. The vertebrae emerged as the most frequently affected sites, followed by the long bones of the lower limbs. *Nocardia asteroides* was identified as the predominant pathogen. Common complications included abscess development and fever, with TMP-SMX being the antimicrobial agent most frequently prescribed. The overall mortality rate across the reported cases was 9.3%.

The scarcity of documented cases of osteomyelitis caused by *Nocardia* spp. in the literature renders it difficult to define accurate epidemiological patterns for this infection [7]. In the present review, most patients were male, with a median age of 54 years. Notably, the majority of reported cases originated from North America, followed by Asia. The higher prevalence of the infection in North America, and especially, the United States (U.S.) may be attributed to better diagnostic capacity, since U.S. hospitals have widespread access to advanced microbiology labs and can more reliably detect *Nocardia* species. Moreover, larger numbers of immunocompromised patients, such as cancer, organ transplant patients, or individuals undergoing immunosuppressive therapies exist in these high-income countries. These conditions increase the susceptibility to opportunistic infections like *Nocardia* [7,54]. In contrast, the lower number of cases observed in Africa, South America, or Oceania may be a result of underdiagnosis due to limited laboratory capacity, since many hospitals in low-resource regions lack access to specialized microbiological techniques like prolonged cultures; *Nocardia* grows slowly and can be missed unless specifically suspected and cultured for up to 2–3 weeks [55]. In rural parts of Africa, South America, and Oceania, patients may also not have timely access to imaging or biopsy, so bone infections may be undiagnosed or only clinically presumed without microbiologic proof [10]. However, the possibility of publication or language bias is high, given that cases from these regions may be reported only in local-language journals not indexed in large medical databases or not published at all due to resource limitations; in contrast, U.S. clinicians and academic centers publish more case reports in English-language journals, which inflates the apparent numbers in literature searches.

First identified by Edmond Nocard in 1888 as the aerobic actinomycete responsible for bovine farcy, the genus *Nocardia* has since undergone continuous taxonomic refinement, with over 50 species currently recognized [6]. *Nocardia* species belong to the actinomycetes group, whose members share similar morphological features despite notable phylogenetic diversity. This species is a genus of aerobic, Gram-positive bacteria that, unlike most other Gram-positive organisms, present on Gram staining as branching, beaded, filamentous rods [56]. These microorganisms are saprophytic, commonly inhabiting soil as well as decomposing plant material; they have occasionally been isolated from various environmental sources including water, garden soil, household dust, and beach sand [2,57]. Characteristically, *Nocardia* spp. is slow-growing, with routine cultures typically requiring 3 to 21 days for visible growth [58]. Infection most often occurs via inhalation, rendering the lungs as the most commonly affected organs, while direct cutaneous inoculation represents the second most common route [2]. The infection can spread from a primary pulmonary or cutaneous site to almost any organ in the body. There is no documented evidence of respiratory transmission from infected animals to humans or of human-to-human spread, although rare cases have raised this possibility [57]. Although up to 10–50% of nocardiosis cases occur in immunocompetent individuals, the infection is generally regarded as opportunistic. *Nocardia asteroides* is the species most frequently linked to human disease, followed by *N. brasiliensis*, which constitutes a common cause of mycetoma in Mexico and South America and is typically associated with localized, cutaneous infections [10,59]. In regions where the pathogen is endemic, nocardiosis should be considered as a possible cause of osteomyelitis, particularly when it follows skin trauma and potential inoculation of the organism.

The precise prevalence of nocardiosis remains uncertain, though older estimates reported 500–1000 cases annually in the U.S. [56]. While up to one third of the infected patients are immunocompetent, the disease is generally considered opportunistic; effective cell-mediated immunity plays a key role in controlling *Nocardia* infections. Significant risk factors include organ transplantation, malignancy, diabetes mellitus, chronic alcoholism, AIDS, and prolonged corticosteroid therapy [2,60]. In accordance with these data, the present review identified immunosuppression as the predominant risk factor for the disease. In immunocompetent individuals, *Nocardia* infection may remain subclinical or resolve spontaneously, whereas in immunocompromised patients, it is often linked to substantial morbidity and mortality [24]. Nocardiosis predominantly affects individuals with impaired cell-mediated immunity. Dissemination of the infection is generally controlled by T-cell-mediated immunity, highlighting the role of impaired cell-mediated responses in the increased susceptibility observed among immunosuppressed patients such as those with HIV/AIDS [61]. Regarding patients with autoimmune diseases, the majority receive long-term corticosteroids or cytotoxic therapies, increasing their susceptibility to a range of opportunistic infections [2]. Moreover, diabetes mellitus is frequently reported in the literature as an immunosuppressive condition. The heightened susceptibility in diabetic patients is thought to result from impairments in both innate and adaptive cell-mediated immune responses [8]. Certain *Nocardia* strains resist oxidative killing, undermining initial host defenses. This may contribute to the difficulty normal hosts face in controlling infection and further compromises diabetic patients, whose neutrophils already exhibit impaired chemotaxis, phagocytosis, and oxidative killing [62]. This group of patients are also at increased risk for osteomyelitis due to reduced extremity blood perfusion, impaired wound healing, and peripheral neuropathy, which can allow minor trauma to go unnoticed [7].

An additional patient group at increased risk of *Nocardia* osteomyelitis are organ transplant patients. The incidence of *Nocardia* infections among solid organ transplant recipients ranges from 0.7% to 3%, occurring most commonly after heart and kidney transplants and less frequently following liver or lung transplantation [63,64]. In the present review, kidneys constituted the most commonly transplanted organ among the infected transplant patients. Organ transplant patients are more susceptible to *Nocardia* infection primarily because they receive immunosuppressive therapies, including prolonged corticosteroid administration, which suppresses cell-mediated immunity. Additionally, the administration of multiple immunosuppressive agents concurrently leads to the cumulative suppression of both innate and adaptive immunity [65]. This induced immunosuppression reduces T-cell function, impairs macrophage activity, and decreases neutrophil response, thus creating an environment where opportunistic infections can disseminate more easily [66]. As demonstrated by a study by Deem et al., effective T-lymphocyte responses, representing adaptive cell-mediated immunity, are crucial for controlling *Nocardia* infections [67]. History of trauma constitutes another common risk factor among patients with *Nocardia* osteomyelitis, and specifically, the second most common risk factor identified in the present study. Traumatic injury, especially open fractures or puncture wounds contaminated with soil or organic matter, can introduce *Nocardia* directly into bones and surrounding tissues [13,23]. A favorable environment for bacterial colonization is created due to the impairment of local immune defenses. Even minor traumas may be responsible for the disease, since they are not noticed promptly, and particularly in immunocompromised patients, allow the infection to establish before intervention [33].

The clinical presentation of *Nocardia* osteomyelitis is nonspecific, making it difficult to distinguish from other bone infections. Typically, the majority of cases present with localized pain and swelling, low-grade systemic symptoms, fever, elevated inflammatory indices, and discomfort aggravated by ambulation or activity [68]. The clinical manifestations depend on the particular bone that is involved; for instance, in cases of pelvic osteomyelitis, the pain can extend to the suprapubic, perineal, or inguinal areas and may even manifest as abdominal pain with guarding, resembling acute appendicitis [68]. The involvement of the skull carries the risk of serious complications, including cavernous sinus thrombosis and abducens nerve palsy, due to its anatomical proximity to vital structures [69]. Other severe clinical complications comprise abscess formation, identified as the most common complication in this review, in addition to pathological fractures or ulceration [22,25]. The duration of symptoms may extend to years before the condition is accurately diagnosed [46]. The disease rarely involves small or flat bones; it is predominantly transmitted hematogenously, accounting for its predilection for long bones, which are more vascularized and metabolically active [70]. A single osseous site is typically affected, with the vertebrae and the longer bones of the lower extremities most often involved. Nonetheless, multifocal osteomyelitis is not uncommon. A distinctive finding of the present review was that lower respiratory infection was not present in all included cases; only 20% of the included patients presented with *Nocardia* pneumonia. Given that *Nocardia* infection is characterized by diverse clinical manifestations, the possibility of misdiagnosis remains elevated [17]. *Nocardia* osteomyelitis may mimic a bone tumor or an abscess caused by other pathogens, occasionally presenting as a pathological bone fracture or the presence of fistulas. Such nonspecific presentations may lead clinicians to initially attribute symptoms to common infectious or inflammatory conditions, especially in the absence of microbiological confirmation. This aligns with the findings of the present narrative review, in which the misdiagnosis rates were quite high; specifically, 50% were misdiagnosed with other infections, while 25% were diagnosed with immune-mediated diseases such as polymyalgia rheumatica [6]. These patterns indicate how overlapping radiological and clinical features may easily divert the diagnostic approach away from *Nocardia*.

The radiological features of *Nocardia* osteomyelitis are nonspecific, typically presenting as lytic, well-demarcated lesions, which may or may not exhibit periosteal reactions [20]. These imaging findings can be mistaken for neoplastic lesions or infections caused by other microorganisms such as *Actinomyces* spp. or *Mycobacteria* species [44,53]. Definitive diagnosis is established by identifying the causative organism by direct microscopy and the culture of biological samples from abscess drainage, aspiration, or open bone biopsy [11,35]. Modified Ziehl–Neelsen staining with 1% sulfuric acid reveals *Nocardia* species as filamentous, branching, and beaded acid-fast bacilli [20]. *Nocardia* is a slow-growing organism, usually taking around one week in culture to develop characteristic colonies, although in some cases, full growth may require two to three weeks of incubation, thereby prolonging the time to diagnosis [71]. Failure to detect the pathogen can be attributed to the inadequate duration of culture. Moreover, in cases where slow-growing bacteria such as *Nocardia* coexist with rapidly proliferating species, the latter can quickly dominate culture media, masking or completely suppressing the growth of *Nocardia* colonies. This competitive overgrowth not only delays recognition of the true pathogen but may also mislead clinicians toward an alternative, incorrect diagnosis. Notably, cultures of patients with chronic osteomyelitis frequently yield negative results [43]. In order to enhance culture positivity rates, the cessation of antimicrobial therapy for a minimum of 1–2 weeks before obtaining bone tissue samples is recommended. Also, in cases of implant-associated chronic osteomyelitis, accurate microbiological assessment is further supported by collecting specimens from at least five distinct sites surrounding the implant [72]. However, given the rarity of the disease, current knowledge relies largely on isolated case reports and small series; the absence of multicenter registries and systematic data collection impedes comprehensive understanding of diagnostic challenges in *Nocardia* osteomyelitis.

Further species-level identification can be achieved using polymerase chain reaction (PCR) or 16s rRNA sequencing. Gene sequencing is considered as the gold standard for *Nocardia* spp. identification, with 16s rRNA analysis offering high sensitivity and specificity for the molecular detection and species-level characterization of *Nocardia* [73]. In a case series by Li et al., this method was successfully applied to identify three cases of bone infection due to *Nocardia* [43]. More recently, MALDI- TOF MS has emerged as a faster, accurate, and more economical alternative to gene sequencing approaches [20]. The MALDI-TOF MS technique enables rapid and reliable identification. In a case report by Raszka et al., the causative bacterium was successfully identified even from the fifth day of cultivation with this method [21]. Most commercial databases often lack sufficient spectra for rarer *Nocardi*a species, such as *N. cyriacigeorgica,* limiting the identification rates compared with rRNA sequencing. Adjusting the identification score cut-offs improves the accuracy; scores of 1.7–1.9 enable correct genus or species-level identification in 83–90% of cases, with minimal misidentification rates [21,74]. In recent years, metagenomic next-generation sequencing (mNGS) has emerged as a powerful genomics-based approach for infectious disease diagnosis, offering high-precision detection of diverse pathogens and proving especially valuable in complex or critical cases where conventional methods are limited [75,76]. Generally, clinicians conducting microbiological testing are required to possess a substantial level of expertise in order to identify the pathogen promptly and effectively.

The antimicrobial resistance profile of *Nocardia* spp. is of particular interest, given the high misdiagnosis rates, and demonstrates considerable variability, as defined by the CLSI M24 breakpoints [77]. Determination of the pathogen’s susceptibility is challenging due to its slow growth [25]. In the present review, E-test and disk diffusion were the main methods to determine antimicrobial resistance; however, very few cases described the precise method applied. Synergistic activity against *Nocardia* has been demonstrated with TMP–SMX, which is now considered the first-line therapy. In a study of 552 clinical isolates collected from six major medical referral centers in the U.S. between 2005 and 2011, resistance to TMP–SMX and/or sulfamethoxazole was observed in only 2% of isolates [78]. Nonetheless, TMP–SMX resistance is increasingly reported, probably due to its extensive use in *Pneumocystis jirovecii* prophylaxis among immunocompromised individuals [17]. In the present review, resistance to TMP-SMX was relatively low and only observed in 2 out 23 reported cases. Results regarding resistance to empiric agents, such as cephalosporins, are controversial. Previous studies support that most isolates (88–100%) remain susceptible to third-generation cephalosporins, while susceptibility to imipenem is limited to 20–30% of isolates; others found that some species, such as *N. farcinica*, are resistant to these commonly used antibiotics [79]. In our study, resistance to cephalosporins was higher compared with carbapenems. Additionally, *Nocardia* can develop antibiotic resistance during therapy by transforming into L-forms, which lack cell walls [8]. Consequently, treatment failure may warrant repeat culturing to determine an updated antimicrobial susceptibility profile for therapy guidance.

Management of *Nocardia* osteomyelitis typically involves a combination of surgical and medical approaches, including thorough surgical debridement of the infected bone to remove necrotic tissue, along with the administration of appropriate parenteral antibiotics to eradicate the pathogen and prevent recurrence [10,80]. Antimicrobial therapy, regardless of site, should be guided by susceptibility testing and empirical antibiotic coverage with at least two antimicrobials should be administered given the variable pathogen’s resistance patterns [8,20]. First-line treatment for nocardiosis consists of TMP-SMX, dosed at 5–10 mg/kg/of the trimethoprim component. Alternative therapeutic options include third-generation cephalosporins, carbapenems, amikacin, and linezolid [81]. In cases involving TMP–SMX resistance or allergy, the use of amikacin in combination with imipenem represents a preferred therapeutic approach [57]. Although the precise duration of therapy has not been firmly established, extended courses of antibiotics are typically employed. The duration of therapy is determined by both the site of infection and the patient’s immune status. Non-immunocompromised individuals typically require antibiotic therapy for at least six to twelve months [10]. Co-existing pulmonary nocardiosis in immunocompetent individuals generally requires a minimum of six months of treatment, whereas dissemination to the CNS necessitates therapy for at least twelve months [2]. When bone involvement occurs, surgical debridement is essential to remove necrotic and damaged tissue and is typically combined with a minimum of four months of antibiotic therapy [7,20]. Surgical drainage of abscesses can also significantly facilitate recovery. In accordance with these recommendations, the majority of the patients in this review successfully underwent surgical procedures alongside antimicrobial treatment. Overall, the clinical outcomes were positive in most patients, with a survival rate of 90.74%. Deaths were uncommon and primarily associated with severe comorbidities.

This study was subjected to certain limitations. The literature search may not have identified all pertinent studies on epidemiology and mortality, as some publications could have been missed due to the search strategy employed. Our analysis was confined to case reports and case series that depend on precise documentation to ensure accuracy. Furthermore, a number of studies reported insufficient data, limiting our analysis to the data available. Consequently, only studies with complete datasets were included in the findings presented. Finally, research published in languages other than English was excluded from this review. In addition, the narrative approach carries methodological limitations; there was no PROSPERO registration or adherence to PRISMA guidelines and no formal risk-of-bias assessment. The search and synthesis were not exhaustive, and quantitative meta-analysis was not feasible. These factors should be considered when interpreting the findings.

## 5. Conclusions

This narrative review provides a comprehensive overview of the epidemiology, clinical features, microbiological characteristics, therapeutic approaches, and outcomes associated with osteomyelitis caused by *Nocardia* spp., underscoring its pathogenic significance. Among the species reported, *N. asteroides* emerged as the predominant pathogen, with vertebrae involvement most frequently observed. Although standardized treatment guidelines are lacking, TMP-SMX was widely utilized. Clinical outcomes were closely linked to the host’s immune status, emphasizing the importance of the early initiation of antimicrobial and surgical treatment for successful management. Due to the opportunistic behavior of *Nocardia* spp. and the often nonspecific or atypical nature of its presentation, heightened clinical and laboratory vigilance is essential for timely and accurate diagnosis. In this regard, *Nocardia* spp. should always be considered in the case of chronic or atypical osteomyelitis, while early microbiological sampling and the administration of prolonged antibiotic therapy remain critical to achieving favorable outcomes. Despite inherent limitations, this review highlights the necessity for well-designed longitudinal and controlled studies to deepen the understanding of *Nocardia* osteomyelitis and underscores the need for multicenter, prospective studies to establish robust, evidence-based treatment guidelines.

## Figures and Tables

**Table 1:** Characteristics of patients with *Nocardia* osteomyelitis.

Demographics	All Patients (*n* = 55) *	Survived (*n* = 49) *	Died (*n* = 5) **
Age, years, median	54	54	43
Male gender, *n* (%)	36 (65.5)	32 (65.3)	4 (80)
Risk factors			
Immunosuppression, *n* (%)	12 (21.8)	10 (20.4)	2 (40)
Trauma, *n* (%)	10 (18.2)	10 (20.4)	0
Recent surgery, *n* (%)	7 (12.7)	7 (14.3)	0
Recent antibiotic treatment, *n* (%)	7 (12.7)	7 (14.3)	0
Organ transplantation, *n* (%)	5 (9.1)	5 (10.2)	0
Diabetes mellitus, *n* (%)	4 (7.3)	3 (6.1)	1 (20)
Autoimmune syndrome, *n* (%)	4 (7.3)	4 (8.2)	0
No predisposing factors, *n* (%)	16 (29.1)	15 (30.6)	1 (20)
Clinical characteristics			
Abscess, *n* (%)	31 (56.4)	26 (53.1)	5 (100)
Fever, *n* (%)	25 (45.5)	22 (44.9)	3 (60)
Fistula, *n* (%)	16 (29.1)	15 (30.6)	1 (20)
Treatment			
TMP-SMX, *n* (%)	44/54 (81.5)	40 (81.6)	4 (80)
Cephalosporins, *n* (%)	16/54 (29.6)	15 (30.6)	1 (20)
Carbapenems, *n* (%)	15/54 (27.8)	14 (28.6)	1 (20)
Aminoglycosides, *n* (%)	11/54 (20.4)	10 (20.4)	1 (20)
Tetracyclines, *n* (%)	8/54 (14.8)	8 (16.3)	0
Combination of antibiotics, *n* (%)	45/54 (83.3)	41 (83.7%)	4 (80)
Outcomes			
Deaths due to infection, *n* (%)	3/54 (5.6)	NA	NA
Deaths overall, *n* (%)	5/54 (9.3)	NA	NA

TMP-SMX: Trimethoprim-sulfamethoxazole; NA: not applicable; * Data are among the number of patients mentioned on top unless otherwise described, ** Data on survival were not reported in one patient.

**Table 2:** Antimicrobial resistance rates.

Antibacterial Agent	Number of Patients	Resistance (%)
Quinolones	6/12	50
Aminopenicillins	6/13	46.2
Cephalosporins	4/11	36.4
Macrolides	5/14	35.7
Aminoglycosides	5/20	25
Penicillin	1/5	20
Amoxicillin-clavulanic	2/11	18.2
Tetracyclines	2/11	18.2
Carbapenems	3/17	17.6
TMP-SMX	2/23	8.7
Linezolid	1/16	6.3

TMP-SMX: Trimethoprim-sulfamethoxazole.

## Data Availability

Not applicable.

## Abbreviations

The following abbreviations are used in this manuscript:

TMP-SMX	Trimethoprim-sulfamethozaxole
CNS	Central nervous system
MALDI-TOF MS	Matrix-assisted laser desorption/ionization time-of-flight mass spectrometry
UTI	Urinary tract infection
